# A Genome-Wide Association Study of the Protein C Anticoagulant Pathway

**DOI:** 10.1371/journal.pone.0029168

**Published:** 2011-12-28

**Authors:** Georgios Athanasiadis, Alfonso Buil, Juan Carlos Souto, Montserrat Borrell, Sonia López, Angel Martinez-Perez, Mark Lathrop, Jordi Fontcuberta, Laura Almasy, José Manuel Soria

**Affiliations:** 1 Unit of Genomics of Complex Diseases, Research Institute, Hospital de la Santa Creu i Sant Pau, Barcelona, Spain; 2 Department of Genetics and Development, University of Geneva, Geneva, Switzerland; 3 Haemostasis and Thrombosis Unit, Department of Hematology, Hospital de la Santa Creu i Sant Pau, Universitat Autònoma de Barcelona, Barcelona, Spain; 4 Centre National de Génotypage, Évry, France; 5 Department of Population Genetics, Southwest Foundation for Biomedical Research, San Antonio, Texas, United States of America; Universite de Montreal, Canada

## Abstract

The *Protein C anticoagulant pathway* regulates blood coagulation by preventing the inadequate formation of thrombi. It has two main plasma components: protein C and protein S. Individuals with protein C or protein S deficiency present a dramatically increased incidence of thromboembolic disorders. Here, we present the results of a genome-wide association study (GWAS) for protein C and protein S plasma levels in a set of extended pedigrees from the *Genetic Analysis of Idiopathic Thrombophilia* (GAIT) Project. A total number of 397 individuals from 21 families were typed for 307,984 SNPs using the Infinium® 317 k Beadchip (Illumina). Protein C and protein S (free, functional and total) plasma levels were determined with biochemical assays for all participants. Association with phenotypes was investigated through *variance component analysis*. After correcting for multiple testing, two SNPs for protein C plasma levels (rs867186 and rs8119351) and another two for free protein S plasma levels (rs1413885 and rs1570868) remained significant on a genome-wide level, located in and around the *PROCR* and the *DNAJC6* genomic regions respectively. No SNPs were significantly associated with functional or total protein S plasma levels, although rs1413885 from *DNAJC6* showed suggestive association with the functional protein S phenotype, possibly indicating that this locus plays an important role in protein S metabolism. Our results provide evidence that *PROCR* and *DNAJC6* might play a role in protein C and free protein S plasma levels in the population studied, warranting further investigation on the role of these loci in the etiology of venous thromboembolism and other thrombotic diseases.

## Introduction

The *protein C anticoagulant pathway* is an important physiological mechanism that regulates blood coagulation. It prevents the inadequate formation of thrombi and has two main plasma components: protein C and protein S.

Protein C (PC) is a vitamin K-dependent serine protease, which acts as an anticoagulant by inactivating activated Factors V (FVa) and VIII (FVIIIa). PC is activated by the thrombin/thrombomodulin complex on the surface of endothelial cells, where it binds with endothelial PC receptor (EPCR) [1], [2]. EPCR also circulates in a soluble form (sEPCR) with similar affinity to both PC and activated PC (APC). Moreover, sEPCR acts as an inhibitor of APC [3].

Protein S (PS) is also a vitamin K-dependent anticoagulant plasma protein. It has no enzymatic activity, but acts as a cofactor to activated PC in the inactivation of FVa and FVIIIa. Moreover, PS is a cofactor for tissue factor pathway inhibitor (TFPI) for inhibiting FXa [4]. PS circulates either as a free molecule (fPS; ∼40% of the total PS) or as a complex with the C4b-binding protein (C4BP-PS; ∼60% of the total PS) [5]. Until recently, it was thought that only fPS had cofactor activity; however, now there is growing evidence that the C4BP-PS complex participates directly in FVa and FVIIIa inactivation [6].

Individuals with PC or PS deficiency present a dramatically increased incidence of thromboembolic disorders [7]. Many of the mutations that cause these deficiencies are located in and around the structural genes of PC and PS (*PROC* and *PROS1* respectively) [8]–[10]. However, a high proportion of families with PC or PS deficiency have no mutations in these genes [11]. Moreover, several polymorphisms in the promoter of the *PROC* gene account for a mere ∼6% of the quantitative variation of PC levels [12]. These observations suggest that the genetic mechanisms underlying PC and PS plasma levels are still largely unknown and that more loci, other than the two structural genes, are involved in the variability of these traits.

A genome-wide linkage analysis using the data from the family-based GAIT Project was performed to find novel loci affecting PC and PS plasma levels [13], [14]. The analysis showed that genotypic variation in the *PROC* and *PROS1* genomic regions is not a primary determinant of the quantitative variation of PC and PS plasma levels. Rather, PC levels showed significant linkage with chromosomal region 16q23. This region contains a candidate gene, *NQO1* coding for NAD(P)H:dehydrogenase quinone 1, involved in vitamin K metabolism [13]. In addition, there was strong evidence of linkage between chromosomal region 1q32 and fPS plasma levels. Interestingly, this region contains the genes that code for the α and β chains of the C4b-binding protein (*C4BPA* and *C4BPB*) [14]. Moreover, using a tagSNP approach, the Cardiovascular Health Study (CHS) reported that polymorphism rs867186 from the gene that codes for EPCR (*PROCR*) was associated with higher levels of circulating PC antigen and that polymorphism rs1878672 from the *IL10* gene was associated with higher fPS levels [15].

More recently, a genome-wide association scan for loci affecting PC plasma levels in a large sample of patients and controls of European descent identified three novel loci (*GCKR*, *EDEM2* and *BAZ1B*), together with two already known (*PROC* and *PROCR*) [16]. In addition, a genome-wide linkage analysis reported that a quantitative trait locus in chromosomal region 20q11 (including genes *FOXA2*, *THBD* and *PROCR*) influences PC levels in one extended family from the GENES study [17], [18]. A subsequent study revealed that *PROCR* haplotype 3 and a SNP from *FOXA2* (rs1055080) were associated with PC levels in this family, but only *PROCR* haplotype 3 was associated also with plasma levels in healthy individuals [19].

The top (i.e., showing the most significant associations) SNPs from the five genes found in the aforementioned genome-wide association scan explained only a fraction (28.2%) of the variance in PC plasma levels [16]. Since previous studies postulated that ∼50% of the phenotypic variation in PC plasma levels is caused by the additive effect of genes [20] the discovery of more loci is tenable. Bearing this in mind, we carried out the first GWAS that encompasses the two main components of the *protein C anticoagulant pathway* (PC and functional, free and total PS levels). The objective of this work was to search for SNPs that influence PC and PS plasma levels and potentially increase the risk of venous thrombosis. We were successful in identifying such loci.

## Methods

### Ethics Statement

The Institutional Review Board of the Hospital de la Santa Creu i Sant Pau approved all protocols used in the GAIT Project and participants gave their informed consent, in compliance with the Declaration of Helsinki.

### The GAIT Project: a brief description

The GAIT Project included 397 individuals from 21 extended Spanish families (mean pedigree size = 19) [20]–[21]. Twelve of these families were selected on the basis of a proband with idiopathic thrombophilia, whereas the remaining nine families were selected randomly. Age ranged from <1 to 88 years (mean = 37.7) and male to female sex ratio was 0.85.

### Plasma measurements

PC plasma concentrations were measured by a biochemical analyzer (CPA Coulter, Coulter Corp) using chromogenic methods from Chromogenix. Functional PS (funcPS) was assayed with the STA automated coagulometer (Boehringer Mannheim) and determined with a Diagnostica Stago kit. fPS and total protein S (free+C4b–bound) were assayed with an ELISA-based commercial kit (Diagnostica Stago). To reduce experimental error, each assay was performed twice and the average value was calculated for each participant. Intra- and inter-assay coefficients of variation were between 2% and 6%.

### Genotypic determinations and data cleaning

A genome-wide set of 307,984 SNPs was typed in all of the participants using the Infinium® 317 k Beadchip on the Illumina platform (San Diego, CA, USA). Genotype imputation was performed with Merlin [22] to avoid missing values and all genotypes were checked for Mendelian inconsistencies. In addition, any SNP with call rate<95%, MAF<0.025 or failing to fit Hardy-Weinberg proportions taking into account multiple testing (p<5×10^−7^) was removed from the study. In total, 24,547 SNPs failed to pass the data cleaning criteria, leaving a set of 283,437 SNPs for further analysis.

### Statistical analysis

Association with phenotypes was investigated through *variance component analysis* that takes into account the family relationships among individuals. The quantitative phenotype (*y*) was modeled as a linear function of the genetic effect of a SNP (*snp*), the polygenic effect (*g*) and a random environmental deviation (*e*):
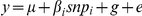



The covariance among phenotypic values (*Ω*) was modeled using the kinship coefficient matrix (*Φ*) derived from the family structure:
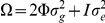
whereby 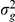 and 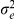 are the variances of the polygenic and environmental effects and *I* is the identity matrix.

The analysis was performed with the SOLAR v4.0 statistical package [23]. Variance component methods present considerable advantages when combined with extended families for the localization of QTLs, as it is now clear that large complex pedigrees have substantially more power per sampled individual than smaller families do [24]–[26]. All plasma phenotypes (PC fPS, funcPS and total PS) were log-transformed and adjusted for age and sex. Measured genotype analysis was used for testing association, assuming an additive genetic model [27]. Finally, the Benjamini – Hochberg (B-H) adjustment [28] was applied to the p-values using the p.adjust function in R and assuming a 10% false discovery rate.

## Results

PC, fPS, funcPS and total PS plasma levels in the GAIT sample have been comprehensively described elsewhere [13], [14]. In brief, PC plasma levels ranged from 37% to 198% those of healthy donors, with a mean value of 118.3% (standard deviation = 19.5%) adjusted for age and gender. In addition, fPS plasma levels ranged from 54% to 166% those of healthy donors and the mean age-adjusted fPS value was 109.4% for men (standard deviation = 21.3%) and 89.2% for women (standard deviation = 18.0%). Finally, mean funcPS and total PS plasma levels were 96.5% (range: 30%–188%; standard deviation = 21.7%) and 101.5% (range: 60%–176%; standard deviation = 20.7%) those of healthy donors respectively.

**Figure 1:**
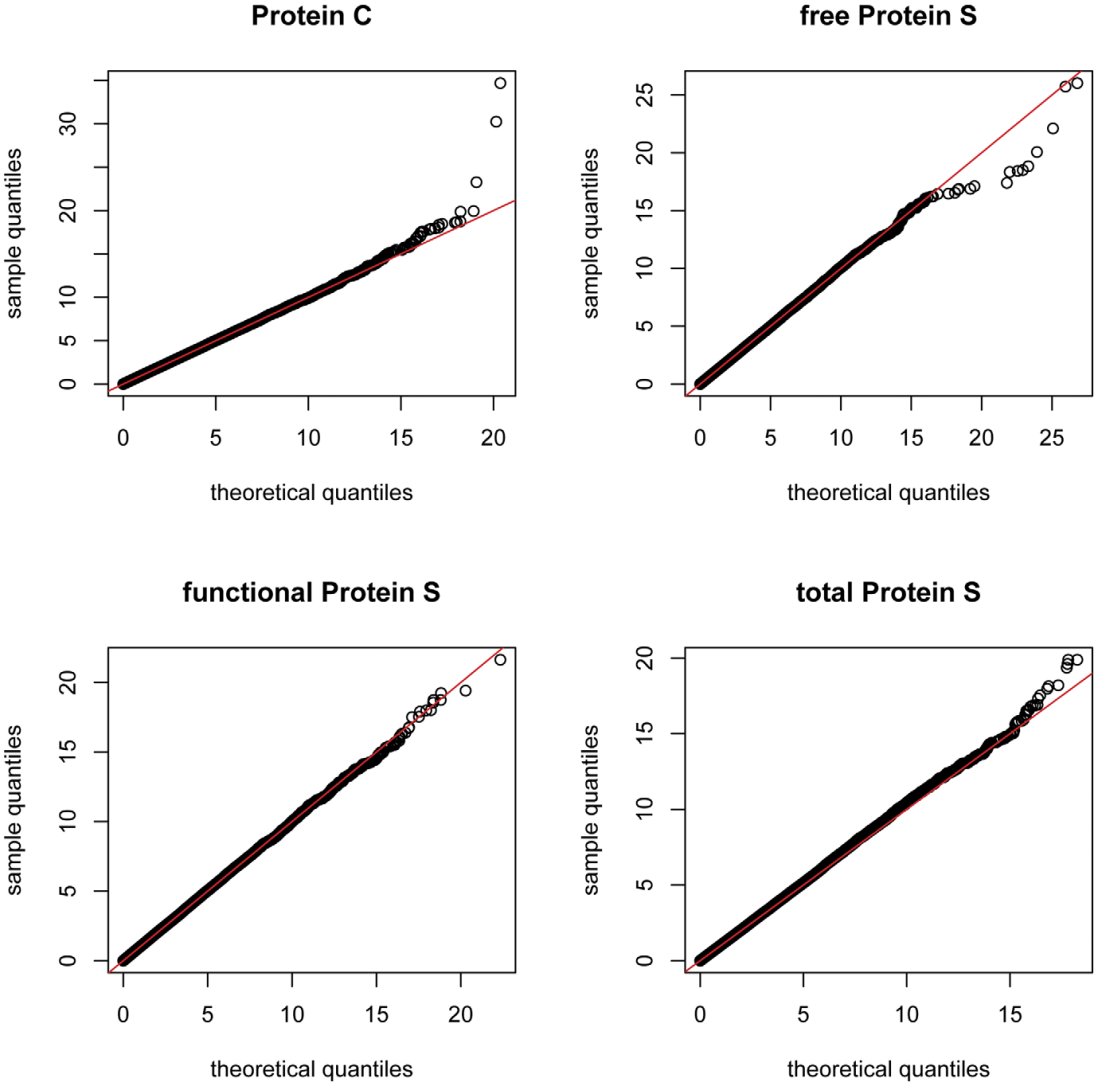
Quantile-quantile plots of theoretical (x-axis) vs. experimental (y-axis) *χ^2^* statistics. Each plot represents one of the four plasma phenotypes (PC, fPS, funcPS and total PS). The red lines (y = x) correspond to equal theoretical and experimental distributions.

Table 1 shows the top SNP associations for each of the four phenotypes (PC, funcPS, fPS and total PS plasma levels). In all plasma phenotypes, association statistics followed the expected *χ^2^* distribution under the null hypothesis of no association (Figure 1). From the 283,437 SNPs that were tested, two SNPs for the PC plasma levels (rs867186 and rs8119351) and another two for the fPS plasma levels (rs1413885 and rs1570868) remained significant on a genome-wide level, after applying the B-H adjustment.

**Table 1:** Top ten SNP associations for PC, fPS, funcPS and total PS plasma levels.

Phenotype	SNP	Location	*χ^2^*	p-values	Gene
**PC**	**rs867186**	**Chr20:33228215**	**34.69**	3.87×10^−09^*	***PROCR***
**PC**	**rs8119351**	**Chr20:33218066**	**30.24**	3.81×10^−08^*	***PROCR***
PC	rs11906160	Chr20:33029416	23.26	1.41×10^−06^	*MYH7B*
PC	rs13230047	Chr7:36927845	19.93	8.02×10^−06^	*ELMO1*
PC	rs1006973	Chr14:51146839	19.86	8.33×10^−06^	*FRMD6*
PC	rs6060239	Chr20:33158241	18.74	1.50×10^−05^	*C20orf31*
PC	rs6691481	Chr1:19216466	18.67	1.56×10^−05^	*RBAF600*
PC	rs884608	Chr9:36490359	18.60	1.61×10^−05^	*MELK*
PC	rs915664	Chr6:30902596	18.46	1.74×10^−05^	*DDR1*
PC	rs780873	Chr12:114680164	18.35	1.84×10^−05^	*THRAP2*
**fPS**	**rs1413885**	**Chr1:65588247**	**26.02**	3.37×10^−07^*	***DNAJC6***
**fPS**	**rs1570868**	**Chr1:65603196**	**25.72**	3.94×10^−07^*	***DNAJC6***
fPS	rs12086738	Chr1:65580000	22.10	2.58×10^−06^	*DNAJC6*
fPS	rs2137111	Chr15:75638590	20.07	7.48×10^−06^	*LRRN6A*
fPS	rs2439430	Chr15:64704898	18.83	1.43×10^−05^	*LCTL*
fPS	rs7983232	Chr13:21635599	18.50	1.70×10^−05^	*FGF9*
fPS	rs10489924	Chr1:99231562	18.43	1.76×10^−05^	*PAP2*
fPS	rs2375699	Chr1:65580869	18.34	1.85×10^−05^	*DNAJC6*
fPS	rs1878449	Chr4:155107054	17.39	3.04×10^−05^	*SFRP2*
fPS	rs4295666	Chr8:22618037	17.11	3.53×10^−05^	*PEBP4*
funcPS	rs13130255	Chr4:43232357	21.63	3.31×10^−06^	*KCTD8*
funcPS	rs3829183	Chr10:70793644	19.41	1.06×10^−05^	*HK1*
funcPS	rs1013719	Chr7:10521275	19.24	1.15×10^−05^	*NDUFA4*
funcPS	rs1106523	Chr2:241900212	18.73	1.50×10^−05^	*SEPT2*
funcPS	rs6724257	Chr2:241871070	18.73	1.50×10^−05^	*HDLBP*
funcPS	rs425459	Chr17:47409200	18.55	1.66×10^−05^	*CA10*
funcPS	rs2712001	Chr4:38823444	18.01	2.20×10^−05^	*KLHL5*
funcPS	rs763773	Chr6:144434079	17.99	2.22×10^−05^	*SF3B5*
funcPS	rs6921460	Chr6:144439852	17.90	2.33×10^−05^	*SF3B5*
funcPS	rs1413885	Chr1:65588247	17.53	2.82×10^−05^	*DNAJC6*
Total PS	rs1401543	Chr3:137070501	19.88	8.23×10^−06^	*PPP2R3A*
Total PS	rs1607504	Chr3:137076926	19.88	8.23×10^−06^	*PPP2R3A*
Total PS	rs2523674	Chr6:31544768	19.60	9.53×10^−06^	*HCP5*
Total PS	rs7648592	Chr3:137057769	19.36	1.08×10^−05^	*PPP2R3A*
Total PS	rs6762218	Chr3:137064983	18.20	1.99×10^−05^	*PPP2R3A*
Total PS	rs7029526	Chr9:119162623	18.13	2.06×10^−05^	*ASTN2*
Total PS	rs7095665	Chr10:87294479	17.96	2.25×10^−05^	*GRID1*
Total PS	rs1372328	Chr9:118524349	17.53	2.82×10^−05^	*ASTN2*
Total PS	rs2723603	Chr11:133174597	17.32	3.16×10^−05^	*SPATA19*
Total PS	rs10241576	Chr7:37825948	16.90	3.93×10^−05^	*TXNDC3*

An asterisk in the p-value indicates significance after B-H adjustment for multiple comparisons.

Polymorphism rs867186 is located in the *PROCR* gene and is responsible for a non-synonymous substitution in the amino acid chain of EPCR (S219G), whereas polymorphism rs8119351 is intergenic, located at ∼10 Kbp upstream from rs867186, with no apparent function. Each copy of minor allele from rs867186 (G) and rs8119351 (A) seems to increase PC plasma levels by 0.845 and 0.812 standard deviations and to explain 10.27% and 9.56% of the variance in PC plasma levels respectively (Table 2). However, these observations are not independent as both SNPs belong to the same LD block (D′ = 0.99; r^2^ = 0.91; p = 6.74×10^−15^). On the other hand, both significant SNPs for the fPS plasma levels (rs1413885 and rs1570868) are intronic, located in the *DNAJC6* gene, and have no known function. In this case, each copy of minor allele from rs1413885 (C) and rs1570868 (T) seems to increase fPS plasma levels by 0.428 and 0.415 standard deviations and to explain 6.24% and 7.53% of the variance in fPS plasma levels respectively (Table 2). Again, these observations are not completely independent as the two SNPs present significant LD (D′ = 0.78; r^2^ = 0.44; p = 1.36×10^−14^). It is also important to note that two more SNPs from the *DNAJC6* genomic region (rs12086738 and rs2375699) also showed suggestive association with fPS plasma levels (Table 1).

**Table 2:** Summary of the four statistically significant SNPs on a genome-wide level.

	rs867186	rs8119351	rs1413885	rs1570868
Position	20q11.22	20q11.22	1p31.3	1p31.3
Genomic region	*PROCR*	*PROCR*	*DNAJC6*	*DNAJC6*
Function	Missense	Intergenic	Intronic	Intronic
Alleles (major/minor)	*A/G*	*G/A*	*T/C*	*C/T*
MAF(1)	0.077	0.074	0.322	0.391
β(2)	0.845	0.812	0.428	0.415
R^2^ (3)	10.27	9.56	6.24	7.53

LD estimates were based on founders alone.

(1) MAF: minor allele frequency based only on founders;

(2) β: effect size on PC (for rs867186 and rs8119351) and PS (for rs1413885 and rs1570868) plasma levels per minor allele (standard deviation scale);

(3) R^2^: proportion of variance explained by each SNP assuming lack of LD.

Although none of the SNPs were significantly associated with funcPS or total PS plasma levels (Table 1), one of the significant SNPs for fPS (rs1413885) also ranked among the top hits for funcPS (p = 2.82×10^−05^), suggesting that *DNAJC6* might be involved in the PS metabolism. Finally, it is also worth noting that four out of five top hits for total PS levels were from the same genomic region (*PPP2R3A*). Although none of these SNPs rose to genome-wide significance levels (p-values between 10^−06^ and 10^−05^) they deserve special attention.

## Discussion

The aim of this study was to shed more light on the genetic mechanisms underlying the *protein C anticoagulant pathway* through a GWAS of the plasma levels of PC, fPS, funcPS and total PS; these levels are strongly involved in the development of thromboembolic disorders.

We were able to detect associations between two tightly linked SNPs from the *PROCR* genomic region (coding for EPCR) and PC plasma levels, also found in previous studies [15], [16], [18], [19]. In this respect, these particular results of ours stand as an independent replication from a family-based perspective. EPCR is an endothelial cell-specific transmembrane protein that is involved in the *protein C anticoagulant pathway* by enhancing the activation rate of PC [29], [30]. Increased levels of sEPCR have been associated with an increased risk of thrombotic events [31], [32]. From the two most significant SNPs we found in the *PROCR* gene, rs867186 is more likely to play a causative role in determining the PC plasma levels, as it is located in exon 4 of the *PROCR* gene and leads to an amino acid change (S219G). More importantly, previous studies have associated S219G with increased risk of venous thromboembolism [33], [34]; moreover, a haplotype including S219G has been associated with increased risk of venous thromboembolism in carriers of (i) Factor V Leiden [35]; (ii) the G20210A mutation in the prothrombin gene [36]; and (iii) other dysfunctional PC variants [32]. It has been proposed that S219G either affects the binding properties of sEPCR or enhances its secretion from the endothelial surface leading to alterations of circulating PC [15].

In addition, we were able also to detect significant associations between two SNPs from a novel candidate gene (*DNAJC6*) and fPS plasma levels, as well as suggestive associations between another two SNPs from *DNAJC6* and the same trait. Interestingly, the most significant SNP for fPS plasma levels (rs1413885) also showed suggestive association with funcPS, underpinning the involvement of *DNAJC6* in different PS traits. The exact function of *DNAJC6* is still unknown; according to the UniProt database (http://www.uniprot.org) the protein coded by *DNAJC6* resembles a tyrosine-protein phosphatase auxilin, an enzyme promoting the uncoating of clathrin-coated vesicles, thus playing a possible role in endocytosis. Endocytosis itself, followed by partial proteolysis, is involved in coagulation, through the molecular modification of FV and FVIII: partially proteolyzed FV exhibits significant procoagulant activity and resistance to activated PC [37]. Thus, a similar mechanism involving *DNAJC6* and fPS is possible, although the validation of this hypothesis would require further investigation.

Even though we were successful in discovering a novel locus for fPS plasma levels (*DNAJC6*) and replicating previous data on PC plasma levels, we did not find any significant associations for funcPS and total PS plasma levels. An *ad hoc* query of the STRING database (http://string.embl.de) for pathways of possible biological relevance involving the top hits from each PS phenotype (as listed in Table 1) gave no evidence of protein–protein interactions.

In a previous linkage study based on the GAIT sample, we reported *NQO1* as a candidate gene affecting variation in PC plasma levels [13]. Further analysis showed that one intronic SNP (rs1437135) from *NQO1* was significantly associated with PC plasma levels [13]. Unfortunately, this SNP was not present in the Illumina chip that we used for our genome-wide association analysis, so no direct comparisons could be made. Nevertheless, another SNP from the *NQO1* genomic region (rs1800566) was included in the Illumina chip. Although this SNP is in full LD with rs1437135, it did not show significant association with PC plasma levels on a genome-wide level (p = 0.061). Moreover, we have reported strong evidence of linkage between chromosomal region 1q32 and fPS plasma levels; interestingly, this region contains two genes of high biological relevance, *C4BPA* and *C4BPB*, coding for the principal binding protein of PS [14]. However, our genome-wide association study found no association between these two genes and any of the PS phenotypes on a genome-wide level.

This is the first time we have evidence that the *PROCR* gene is associated with PC plasma levels in the GAIT sample, as the linkage study performed previously [13] did not identify any linkage between the *PROCR* genomic region and this phenotype [logarithm of the odds (LOD) score = 0.670]. In a similar manner, no linkage was previously found between the *DNAJC6* genomic region and fPS plasma levels (LOD score = 0.089) [14]. Several issues arise from this apparent lack of concordance between genome-wide association and genome-wide linkage studies from the GAIT Project. It is important to note that GAIT is one of the few projects that allow us to perform this comparison.

From the methodological point of view, linkage differs from association in that it is based on the joint transmission of a marker and a functional site from parent to offspring (i.e. co-segregation), rather than on correlation due to LD. In this context, the association approach has difficulty in detecting rare variants through LD with common SNP markers, but such variants can be found by linkage. Thus, an explanation for the failure to detect the same loci in our analysis might be that linkage signals in GAIT might be due to rare variants at those loci, whereas association might be due to more common variants. This observation does not rule out the presence of other variants at those loci with small effect on PC or PS levels and our study does not have enough power to detect such small effects. It is important to note that if we cannot detect the effect of the other QTLs because it is small, this emphasizes our results that *PROCR* and *DNAJC6* are major determinants of PC and PS levels in the Spanish population.

Taken together, the linkage and association analyses we carried out in the context of the GAIT Project are a good example of how rare and common variants underlying the genetic architecture of complex traits, such as PC and PS plasma levels. In addition, it is important to emphasize that no single method or model for studying genetic architecture can be adopted universally. No single method can answer all or even some of the questions without being used in concert with additional approaches.

In summary, our work provides evidence that the *PROCR* and *DNAJC6* loci are involved in the genetic determination of the PC and fPS plasma levels respectively. However, these observations should be further validated by means of functional experiments, especially for the fPS plasma levels, as the function of the *DNAJC6* gene is still unknown.

## References

[1] Walker FJ (1981). Regulation of activated protein C by protein S: the role of phospholipid in factor V inactivation.. J Biol Chem.

[2] Walker FJ, Chavin SI, Fay PJ (1987). Inactivation of factor VIII by activated protein C and protein S.. Arch Biochem Biophys.

[3] Van de Wouwer M, Collen D, Conway EM (2004). Thrombomodulin-protein C-EPCR system: integrated to regulate coagulation and inflammation.. Arterioscler Thromb Vasc Biol.

[4] Hackeng TM, Sere KM, Tans G, Rosing J (2006). Protein S stimulates inhibition of the tissue factor pathway by tissue factor pathway inhibitor.. Proc Natl Acad Sci USA.

[5] Dahlback B (2007). The tale of protein S and C4b–binding protein, a story of affection.. Thromb Haemost.

[6] Maurissen LF, Thomassen MC, Nicolaes GA, Dahlbäck B, Tans G (2008). Re–evaluation of the role of the protein S–C4b binding protein complex in activated protein C–catalyzed factor Va–inactivation.. Blood.

[7] Lane DA, Mannucci PM, Bauer KA, Bertina RM, Bochkov NP (1996). Inherited Thrombophilia: Part 1.. Thromb Haemost.

[8] Gandrille S, Borgel D, Eschwege-Gufflet V, Aillaud M, Dreyfus M (1995). Identification of 15 different candidate casual point mutations and three polymorphisms in 19 patients with protein S deficiency using a scanning method for analysis of the protein S active gene.. Blood.

[9] Reitsma PH, Bernardi F, Doig RG, Gandrille S, Greengard JS (1995). Protein C deficiency: a database of mutations, 1995 update. On behalf of the Subcommittee on Plasma Coagulation Inhibitors of the Scientific and Standardization Committee of the ISTH.. Thromb Haemost.

[10] Franco RF, Reitsma PH (2001). Genetic risk factors of venous thrombosis.. Hum Genet.

[11] Koeleman BP, Reitsma PH, Bertina RM (1997). Familial thrombophilia: a complex genetic disorder.. Semin Hematol.

[12] Spek CA, Koster T, Rosendaal FR, Bertina RM, Reitsma PH (1995). Genotypic variation in the promoter region of the protein C gene is associated with plasma protein C levels and thrombotic risk.. Arterioscler Thromb Vasc Biol.

[13] Buil A, Soria JM, Souto JC, Almasy L, Lathrop M (2004). Protein C levels are regulated by a quantitative trait locus on chromosome 16: results from the Genetic Analysis of Idiopathic Thrombophilia (GAIT) Project.. Arterioscler Thromb Vasc Biol.

[14] Almasy L, Soria JM, Souto JC, Coll I, Bacq D (2003). A quantitative trait locus influencing free plasma protein S levels on human chromosome 1q: results from the Genetic Analysis of Idiopathic Thrombophilia (GAIT) project.. Arterioscler Thromb Vasc Biol.

[15] Reiner AP, Carty CL, Jenny NS, Nievergelt C, Cushman M (2008). PROC, PROCR and PROS1 polymorphisms, plasma anticoagulant phenotypes, and risk of cardiovascular disease and mortality in older adults: the Cardiovascular Health Study.. J Thromb Haemost.

[16] Tang W, Basu S, Kong X, Pankow JS, Aleksic N (2010). Genome–wide association study identifies novel loci for plasma levels of protein C: the ARIC study.. Blood.

[17] Wichers IM, Tanck MW, Meijers JC, Lisman T, Reitsma PH (2009). Assessment of coagulation and fibrinolysis in families with unexplained thrombophilia.. Thromb Haemost.

[18] Tanck MW, Wichers IM, Meijers JC, Büller HR, Reitsma PH (2011). Quantitative trait locus for protein C in a family with thrombophilia.. Thromb Haemost.

[19] Pintao MC, Roshani S, de Visser MC, Tieken C, Tanck MW (2011). High levels of protein C are determined by PROCR haplotype 3.. J Thromb Haemost.

[20] Souto JC, Almasy L, Borrell M, Garí M, Martínez E (2000). Genetic determinants of hemostasis phenotypes in Spanish families.. Circulation.

[21] Souto JC, Almasy L, Borrell M, Blanco-Vaca F, Mateo J (2000). Genetic susceptibility to thrombosis and its relationship to physiological risk factors: the GAIT study Genetic Analysis of Idiopathic Thrombophilia.. Am J Hum Genet.

[22] Abecasis GR, Cherny SS, Cookson WO, Cardon LR (2002). Merlin-rapid analysis of dense genetic maps using sparse gene flow trees.. Nat Genet.

[23] Almasy L, Blangero J (1998). Multipoint quantitative–trait linkage analysis in general pedigrees.. Am J Hum Genet.

[24] Blangero J, Williams JT, Almasy L (2000). Quantitative trait locus mapping using human pedigrees.. Hum Biol.

[25] Blangero J, Williams JT, Almasy L (2001). Variance component methods for detecting complex trait loci.. Adv Genet.

[26] Blangero J, Williams JT, Almasy L (2003). Novel family-based approaches to genetic risk in thrombosis.. J Thromb Haemost.

[27] Boerwinkle E, Chakraborty R, Sing CF (1986). The use of measured genotype information in the analysis of quantitative phenotypes in man. I. Models and analytical methods.. Ann Hum Genet.

[28] Benjamini Y, Hochberg Y (1995). Controlling the false discovery rate: a practical and powerful approach to multiple testing.. J Roy Statist Soc B.

[29] Laszik Z, Mitro A, Taylor FB, Ferrell G, Esmon CT (1997). Human protein C receptor is present primarily on endothelium of large blood vessels: implications for the control of the protein C pathway.. Circulation.

[30] Stearns-Kurosawa DJ, Kurosawa S, Mollica JS, Ferrell GL, Esmon CT (1996). The endothelial cell protein C receptor augments protein C activation by the thrombin-thrombomodulin complex.. Proc Natl Acad Sci U S A.

[31] Uitte de Willige S, Van Marion V, Rosendaal FR, Vos HL, de Visser MC (2004). Haplotypes of the EPCR gene, plasma sEPCR levels and the risk of deep venous thrombosis.. J Thromb Haemost.

[32] Simioni P, Morboeuf O, Tognin G, Gavasso S, Tormene D (2006). Soluble endothelial PC receptor (sEPCR) levels and venous thromboembolism in carriers of two dysfunctional protein C variants.. Thromb Res.

[33] Trégouët DA, Heath S, Saut N, Biron-Andreani C, Schved JF (2009). Common susceptibility alleles are unlikely to contribute as strongly as the FV and ABO loci to VTE risk: results from a GWAS approach.. Blood.

[34] Galanaud JP, Cochery-Nouvellon E, Alonso S, Chauleur C, Mercier E (2010). Paternal endothelial PC receptor 219Gly variant as a mild and limited risk factor for deep vein thrombosis during pregnancy.. J Thromb Haemost.

[35] Medina P, Navarro S, Estellés A, Vayá A, Bertina RM (2005). Influence of the 4600A/G and 4678G/C polymorphisms in the endothelial PC receptor (EPCR) gene on the risk of venous thromboembolism in carriers of factor V Leiden.. Thromb Haemost.

[36] Navarro S, Medina P, Mira Y, Estellés A, Villa P (2008). Haplotypes of the EPCR gene, prothrombin levels, and the risk of venous thrombosis in carriers of the prothrombin G20210A mutation.. Haematologica.

[37] Camire RM, Bos MH (2009). The molecular basis of factor V and VIII procofactor activation.. J Thromb Haemost.

